# Independent Effects of Emotional Expression and Group Membership in the Evaluative Priming Task

**DOI:** 10.1027/1618-3169/a000628

**Published:** 2024-12-12

**Authors:** Emre Gurbuz, Michaela Rohr, Dirk Wentura

**Affiliations:** ^1^ Psychology Department, Saarland University, Saarbrücken, Germany

**Keywords:** emotional expression, group membership, evaluative priming task, face perception

## Abstract

**Abstract:** Research on automatic evaluative responses to faces varying in emotional expression and ethnicity has yielded conflicting results. Some paradigms, like the Approach/Avoidance task, demonstrated interactive evaluation. In contrast, recent studies using the Evaluative Priming Task (EPT) yielded independent effects of expression and ethnicity. One key difference between these paradigms is the task relevance of the faces. In the EPT faces served solely as primes without direct relevance to the task. To examine whether increased task relevance could engender interactive processing in the EPT, we utilized a modified version of the “bona fide pipeline” EPT. In this adaptation, participants categorized the valence of target words succeeding prime faces followed by probe faces. Participants then judged whether the prime and probe faces depicted the same person, thereby adding task relevance to the prime faces. Experiment 1 revealed independent priming effects of emotion and ethnicity. Since error data and inverse efficiency scores provided evidence for an interactive evaluation, we replicated Experiment 1 using a sequential Bayes testing strategy. Experiment 2 confirmed that the effects of emotion and ethnicity remain independent, indicating that increased task relevance did not yield the integrated processing of emotion and ethnicity as initially hypothesized.


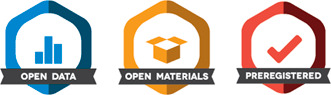


The evaluation of a person is typically influenced by characteristics such as emotional expression, group membership, and other features (age, gender, etc.). However, whether all these features are considered in early and automatic evaluation is debated. The social message account (SMA) proposed and provided evidence that emotion and group membership are processed immediately and affect automatic reactions or motor behaviour to faces interactively (Paulus & Wentura, 2014; Weisbuch & Ambady, 2008).

To outline the underlying assumption: Happiness and fear signal benevolent and malevolent intentions, respectively. However, the interpretation of these emotional signals may differ for a prejudiced observer. For example, consider a White person with a prejudice against Black people. A happy expression from a White person would be interpreted as affiliation, whereas a happy expression from a Black person would be perceived as dominance or mischievousness (Paulus et al., 2016)*.* Conversely, a fearful expression from a White person would be interpreted as a warning signal, whereas a fearful expression from a Black person would be perceived as a signal of submission. Thus, for a prejudiced observer, ingroup happiness and outgroup fear would be associated with a positive social message, and ingroup fear and outgroup happiness would be associated with a negative social message. Empirically, this processing is reflected in an interaction between emotion and group membership, which has already been observed in several studies: Hitherto, evidence for the SMA in early automatic processing has been obtained using several indirect paradigms, namely the Approach/Avoidance task (AAT; Paulus & Wentura, 2014), the extrinsic affective Simon task (EAST; Gurbuz et al., 2023) and the evaluative priming task (EPT; Weisbuch & Ambady, 2008).

However, the accumulated evidence in the EPT has yielded mixed results regarding the early, automatic processing of emotion and group membership (Craig et al., 2014; Paulus & Wentura, 2018; Weisbuch & Ambady, 2008). Namely, Craig et al. (2014) and Paulus and Wentura (2018) found main effects of emotion and group membership, but no interactive pattern. These findings imply that both features are processed, but not interpreted in the assumed way, or that the features are not integrated under certain conditions, raising doubts about the validity of the SMA in fast and early automatic processing conditions.

## The Puzzle of the SMA in the Evaluative Priming Task

In a standard EPT, participants categorize target stimuli based on their valence (i.e., positive or negative). These targets are preceded by briefly presented prime stimuli, which have a valence that is either congruent or incongruent with the target valence. Typically, responses are faster and more accurate when prime and target valences match, and slower and more erroneous in case of incongruency (for a meta-analysis, see Herring et al., 2013). This pattern reflects that the valence of the task-irrelevant prime is processed automatically, subsequently influencing the response to the target (Rohr & Wentura, 2022). Applied to the SMA, primes contain two evaluative features, emotional expression and group membership.

Weisbuch and Ambady (2008) used happy and fearful expressions of ingroup and outgroup (i.e., White and Black) as primes and positive and negative images as targets in the EPT. Results indicated that emotion and group membership of primes interactively influenced evaluative responses: happy ingroup and fearful outgroup faces (relatively) facilitated reactions to positive targets; on the other hand, fearful ingroup and happy outgroup faces (relatively) facilitated reactions to negative targets.^1^

However, subsequent studies that aimed to replicate the interaction pattern between emotion and group membership in the EPT failed. In three experiments, Craig et al. (2014) failed to replicate the findings of Weisbuch and Ambady (2008), using happy and fearful expressions of White and Black individuals as primes and White participants. In all three experiments, instead of the interaction pattern observed by Weisbuch and Ambady (2008), an independent emotion priming effect was observed: happy and fearful primes (relatively) facilitated reactions to positive and negative targets, respectively. An ethnicity priming effect (i.e., White and Black faces relatively facilitating reactions to positive and negative targets, respectively) only occurred when participants were instructed to identify the ethnicity of the prime faces after classifying the target valence. Using White participants and Middle Eastern individuals as the outgroup, Paulus and Wentura (2018) also attempted to replicate Weisbuch and Ambady’s (2008) findings. In three experiments, happy and fearful expressions of White and Middle Eastern faces were used as primes. The results showed independent priming effects of emotion and group (both in the same direction as found by Craig et al., 2014) with no interaction. Thus, the studies that attempted to replicate Weisbuch and Ambady (2008) yielded contrasting results: independent effects of emotion and group but no interactive effects.

## A Potentially Important Difference Between the Paradigms

An important difference between the EPT and the other indirect tasks that have been used to study the EPT is the task relevance of faces: In the AAT (Paulus & Wentura, 2014, Experiment 1) and the EAST (Gurbuz et al., 2023), the face stimuli themselves required a response (although the decisive features of emotion and ethnicity were task-irrelevant). In the EPT, the face stimuli were used as primes and were therefore completely task-irrelevant. It could be argued that this task relevance is necessary for the processes postulated by SMA, as it creates a rudiment of a social communication situation that is missing when the stimulus is completely task-irrelevant.

The present study aimed to increase the task relevance of face primes in the EPT by adopting a version of the bona fide pipeline priming procedure developed by Fazio et al. (1995).

In the original procedure, participants were led to believe that the study involved dual-task performance. They completed an EPT with word targets and were informed that the face primes would be relevant for a subsequent recognition memory task. This procedure required participants to pay attention not only to the targets (as it is the case for the standard priming task) but also to attend to the prime faces, making them task-relevant without raising suspicion about the study’s true aim.

Similarly, in the present study, participants had to pay attention to the prime stimuli because they were used in an additional recognition task. However, the performance in the recognition task was not the main focus: it primarily served as a cover story to enhance the salience of the primes without revealing the primary objective of the experiment. Unlike Fazio and colleagues, who introduced the recognition task as a separate follow-up to the priming task, we embedded the recognition task within the priming task itself (see “Procedure” of Experiment 1). This dual-task setup required participants to first classify the valence of the target (as in the standard priming task) and then determine whether the probe face matched the prime’s identity. This integration made the prime faces task-relevant while minimizing participant suspicions about the true aims of the study.

## Experiment 1

The experiment has been pre-registered at https://aspredicted.org/56R_NYH and the data can be accessed at https://osf.io/4fmev/.

### Method

#### Participants

To detect an effect of *d*_*Z*_ = .40 (see Paulus & Wentura, 2018, for the effect size calculation based on Weisbuch & Ambady, 2008 results) a sample of 84 participants was required. The effective sample was 88 (36 females, 50 males, two undisclosed genders; *Mdn*_age_ = 27 years, range: 18–35). Ninety participants were recruited via Prolific Academic (https://www.prolific.co). Following to pre-registered outlier criteria, data were excluded for participants who reported a non-German mother tongue and/or migrant background (*n* = 1), and who had an error rate of more than 20% in the main task (*n* = 1). The experiment lasted approximately 40 min; participants were paid approximately 5.5 GPB.

#### Design

The study followed a 2 (prime emotion: happy, fearful) × 2 (prime group: White, Middle Eastern) × 2 (target valence: positive, negative) within-participants design.

#### Materials

The prime stimuli consisted of fearful and happy expressions of 10 White and 10 Middle-Eastern men, as used in Paulus and Wentura (2014, 2018). The neutral expression of the same individuals was used for the probe faces. The images were taken from the Radboud Faces Database (Langner et al., 2010), the Amsterdam Dynamic Facial Expression Set (van der Schalk et al., 2011), and our collection (Paulus et al., 2012; for details on the selection procedure see Paulus & Wentura, 2014). The target stimuli consisted of 10 negative and 10 positive words. The words were taken from previous studies (see Gurbuz et al., 2023; Paulus & Wentura, 2018).^2^

#### Procedure

The study was designed using PsychoJS, the JavaScript version of PsychoPy (Peirce et al., 2022), and run on the Pavlovia platform (https://pavlovia.org/, Open Science Tools Ltd.). Participants were recruited through Prolific and automatically redirected to Pavlovia, where the experiment was launched in their web browser. Participation was limited to desktop computers or laptops. To calibrate the display to their screen size, participants adjusted an on-screen image of a credit card to match the actual size of a physical credit (or equivalent) using arrow keys (Morys-Carter, 2021, May 18).

Participants were then introduced to the main tasks, which consisted of three phases: the identity change detection task, the word valence classification task, and the priming task.

##### Identity Change Detection Task (ICD Task)

In the ICD task, each trial began with a centered fixation cross displayed 1,000 ms, followed by a prime face shown for 100 ms. Then a random string of letters appeared for 300 ms before the probe face, which was presented for 2,000 ms. The probe face was either a neutral expression of the same person as the prime (“no change”) or a different person (“change”). Half of the trials featured no change, while the other half presented a neutral expression of an individual from the opposite group to emphasize the group feature. The task was to press the space bar when the first (i.e., prime) and second (i.e., probe) faces belonged to different individuals and to withhold a response when the first and second faces belonged to the same individual. The ICD task consisted of 24 trials, in which a total of 3 White and 3 Middle-Eastern faces displaying both happiness and fear were presented in “change” and “no change” conditions. The faces were different from those used in the priming task.

##### Word Valence Classification Task

In the word valence classification task, each trial began with a centered fixation cross displayed for 1,000 ms, followed by a positive or negative word presented until a response was made or for a maximum of 2,000 ms elapsed. The task was to classify the words as either positive or negative, using the “A” key for “negative” and the “L” key for “positive” responses. The task consisted of 20 trials with each positive and negative word presented once.

##### Priming Task

In the priming task, participants performed both the ICD and word evaluation tasks within each trial. Each trial began with a 1,000 ms fixation cross, followed by a 100 ms prime face. This was followed by a 100 ms blank screen and then a target word, which remained on the screen until a response was made or for a maximum of 2,000 ms elapsed. Participants first classified the target word as positive or negative using the same keys as in the practice word valence classification task. After responding, a neutral probe face was presented with a 1,000 ms delay and remained on the screen for 2,000 ms. The second task was to determine whether the probe face depicted the same individual as the prime face by pressing the space bar for “different” and withholding a response for “same.” The stimulus onset asynchrony between the prime face and target word was 200 ms. In half of the trials, the probe face displayed the same individual as the prime face, while in the other half, it featured an individual from the opposite ethnic group. A new trial started after a 1,000 ms inter-trial interval.

The priming task consisted of 320 trials divided into four blocks, with each block containing 80 trials. In each block, 20 unique faces (10 ingroups and 10 outgroups) appeared a total of 4 times: 2 times with a happy expression (once paired with a negative word and once with a positive word) and 2 times with a fearful expression (once paired with a negative word and once with a positive word). This design ensured a balanced distribution of each face’s emotional expression and word pairing within each block. Consecutive trials avoided repetition of the same prime individual and identical target words. Immediate feedback was not provided after correct or incorrect responses, however, a warning message (i.e., Too slow! Please respond faster!) was presented if participants did not respond within 2,000 ms.

### Results

#### ICD Task

In the ICD task, participants demonstrated an average accuracy of 91.33%.^3^ Thus, participants paid attention to prime faces during the task.

#### Priming Task

Trials with incorrect or missing responses (4.67%) in the priming task were excluded. In addition, trials with RTs that were below 150 ms or above one-and-a-half interquartile ranges above the third quantile within the individual distribution (5.32% of the remaining trials; Tukey, 1977) were excluded.

**Figure 1:**
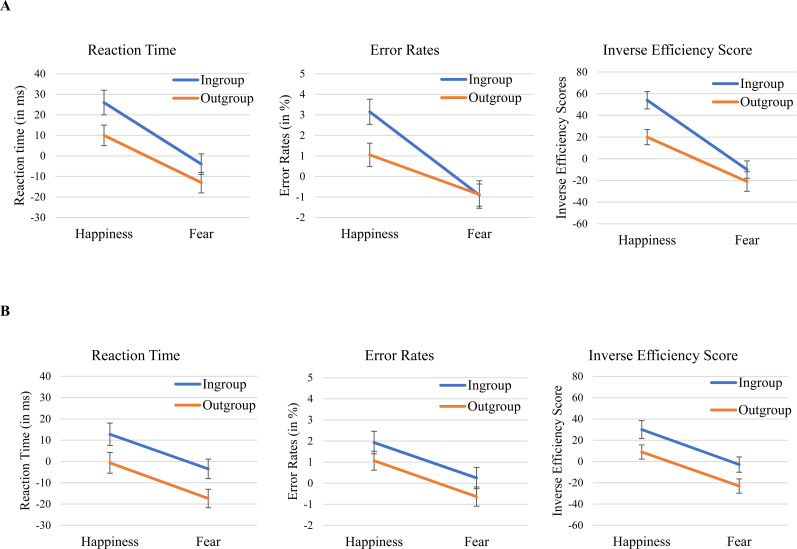
Priming scores for reaction time, error rates, and inverse efficiency scores are presented from left to right across emotional expression and group membership. *Note*. Panel A (top) displays the results for Experiment 1, and Panel B (bottom) presents the results for Experiment 2. The error bars represent the standard error of the means.

Priming scores were calculated by subtracting the mean RTs (error rates) of positive targets from the mean RTs (error rates) of negative targets within each prime category. These difference scores were computed separately for both happy and fearful expressions within ingroup and outgroup categories. Higher positive priming scores indicate relatively more positive evaluations. Table 1 shows RTs, error rates, and IES; Figure 1A shows the mean priming differences.

**Table 1:** Mean response times (in ms), error rates (in %), and inverse efficiency scores as a function of Emotion, Group, and Target Valence of Experiment 1

Prime		Target	
Emotion	Ethnicity	Negative	Positive
Response times			
Happiness	Ingroup	756 (11)	729 (11)
	Outgroup	753 (12)	743 (11)
Fearful	Ingroup	751 (11)	755 (12)
	Outgroup	745 (12)	759 (12)
Error rates			
Happiness	Ingroup	6.16 (.62)	3.01 (.41)
	Outgroup	5.09 (.51)	4.03 (.47)
Fearful	Ingroup	4.26 (.55)	5.17 (.53)
	Outgroup	4.38 (.52)	5.26 (.61)
Inverse efficiency scores (IES)			
Happiness	Ingroup	808 (12)	753 (12)
	Outgroup	795 (13)	775 (12)
Fearful	Ingroup	787 (12)	797 (13)
	Outgroup	782 (14)	803 (13)

##### Response Times

A 2 (prime emotion: happy, fear) × 2 (prime group: ingroup, outgroup) repeated measures ANOVA with RT priming scores as the dependent variable yielded two significant main effects, *F*(1,87) = 59.32, *p* < .001, η_*p*_^2^ = .405, *d*_*Z*_ = .82 for emotion and *F*(1,87) = 9.56, *p* = .003, η_*p*_^2^ = .099, *d*_*Z*_ = 0.33 for group, but no evidence of an interaction *F*(1,87) = 0.69, *p* = .410, η_*p*_^2^ = 0.008, *d*_*Z*_ = 0.09. The priming score for happy faces (*M* = 19 ms, *SD* = 49) was larger than the priming score for fearful faces (*M* = −9 ms, *SD* = 51), and the priming score for ingroup faces (*M* = 11, *SD* = 53) was larger than the priming effect for outgroup faces (*M* = −1, *SD* = 50; see Figure 1A).

##### Error Rates

A corresponding analysis of priming differences in error rates yielded significant effects of emotion *F*(1,87) = 26.06, *p* < .001, η_*p*_^2^ = .230, *d*_*Z*_ = .54, and group *F*(1,87) = 3.18, *p* = .078, η_*p*_^2^ = .035, *d*_*Z*_ = .19. Both main effects corresponded in direction to the effect for priming scores of RTs. Priming differences for happy primes (*M* = 2.10%, *SD* = 5.62) were larger than the priming differences for fearful primes (*M* = −0.89%, *SD* = 5.71), and the priming differences for ingroup faces (*M* = 1.12%, *SD* = 5.75) were larger than the priming differences for Middle Eastern faces (*M* = 0.09%, *SD* = 5.93). The Emotion × Group Interaction was marginally significant, *F*(1,87) = 3.73, *p* = .057, η_*p*_^2^ = .041, *d*_*Z*_ = .21.^4^ The priming score for ingroup happy faces (*M* = 3.15, *SD* = 5.70) exceeded that for Middle-Eastern happy faces (*M* = 1.05, *SD* = 5.37, see Table 1), *t*(87) = 2.46, *p* = .016, whereas ingroup (*M* = −0.91, *SD* = 5.06) and outgroup (*M* = −0.88, *SD* = 6.32) fearful faces were comparable in terms of mean priming scores; *t*(87) = −0.04*, p* = *.*970.

##### Inverse Efficiency Scores (Non-preregistered Analysis)

Closer inspection revealed that RTs (numerically) and error rates showed the same pattern: the group effect was larger for happy faces than for fearful faces, that is, a happy expression from a Middle Eastern was more negative than a happy expression from a White. This discrepancy was not found for fearful expressions.^5^ To get a more conclusive answer to the question of whether the results are best modelled as an interaction pattern, we added an analysis of inverse efficiency scores (IES; Townsend & Ashby, 1978; see Kozlik & Fischer, 2020 for an application to the SMA debate). IES are defined as mean RTs divided by the proportion of correct responses. As processes underlying effects in reaction time tasks may be reflected in RTs or error rates, combining them may provide a clearer picture of the underlying process. Note that Bruyer and Brysbaert (2011) did not unconditionally recommend this measure. However, it might be useful in case of (a) rather low error rates (<10%) and (b) a clear positive correlation of mean RTs and error rates across the conditions (i.e., no evidence of a speed-accuracy tradeoff). Both characteristics are given here (see Table 1).

An analysis with IES priming differences yielded significant effects of emotion, *F*(1,87) = 75.92, *p* < .001, η_*p*_^2^ = .466, *d*_*Z*_ = .93, and group *F*(1,87) = 12.01, *p* < .001, η_*p*_^2^ = .121, *d*_*Z*_ = .37, as well as a marginally Significant Emotion × Group interaction, *F*(1,87) = 3.58, *p* = .062, η_*p*_^2^ = .040, *d*_*Z*_ = .20. The priming score for White happy faces (*M* = 54, *SD* = 71) significantly exceeded that for Middle Eastern happy faces (*M* = 20, *SD* = 69), *t*(87) = 3.81, *p* < .001, *d*_*Z*_ = .41, whereas White (*M* = −11, *SD* = 73) and Middle Eastern (*M* = −21, *SD* = 89) fearful faces were comparable in terms of mean priming scores, *t*(87) = 1.19, *p* = .237, *d*_*Z*_ = .13.

### Discussion

The present study investigated whether the emotional expression and ethnicity of prime stimuli in the EPT independently or interactively influence responses when prime stimuli become task-relevant through a secondary task.

Focusing only on RTs as the dependent variable, independent priming effects of emotion and group were found, replicating previous studies with the EPT (Craig et al., 2014; Paulus & Wentura, 2018). Happy and fearful primes (relatively) facilitated reactions to positive and negative targets, respectively. Moreover, ingroup and outgroup primes (relatively) facilitated reactions to positive and negative targets, respectively. Notably, we found the prejudice-related group effect in an EPT that did not explicitly address the ethnicity of the stimuli, which is consistent with the results of Experiments 2 and 3 by Paulus and Wentura (2018).

However, we obtained a hint of interactive processing of emotion and group when considering errors and the IES. Specifically, the happy expression of outgroup members was significantly less positive than the happy expression of ingroup members. The corresponding difference for fearful expressions was not significant; fearful expressions were negative, irrespective of the ethnicity of the expresser. One might be tempted to wink at these results, given the marginal significance. However, given the mixed and puzzling evidence for the processing of emotion and group membership in the EPT, it may shed light on the underlying processes.

At face value, the valence of happy expressions appears to be influenced by ethnicity, whereas this is not the case for fearful expressions. This outcome is consistent with a weak version of SMA. (A strong version would predict that outgroup fear is even more positive than ingroup fear; see Introduction.)

It is worth noting that Experiment 2 of Paulus and Wentura (2018) already yielded a significant Emotion × Ethnicity Interaction (with priming differences as the dependent variable), which is nearly identical in pattern to the present one.^6^ In the context of two other experiments that did not yield the interaction, Paulus and Wentura (2018) refrained from placing too much emphasis on this result.

To gather more evidence regarding these inconclusive results, we decided to replicate Experiment 1 to determine which of the outcomes would finally manifest in the EPT when primes are made task-relevant by a secondary identity change task.

## Experiment 2

The interaction effect in Experiment 1 for the IES as dependent variable is a small one (*d*_*Z*_ = .20). To interpret a possible null result in Experiment 2, a conventional test strategy would require large power (1 − β = .95) for a conservatively reduced estimate (e.g., *d*_*Z*_ = .15) This would result in a needed sample size of *N* = 580 (α = .05). As this approach would mean an immense sampling effort we decided for sequential hypothesis testing using Bayes Factors (for details, see Schönbrodt et al., 2017) that directly focus on the group effects for happy and fearful faces.

The Bayes factor is a Bayesian model selection metric that quantifies the likelihood of the observed data supporting the null hypothesis (H0) compared to the alternative hypothesis (H1). For example, if H0 and H1 are considered equally probable before data collection, a Bayes factor of BF_10_ = 6 suggests that the data are six times more likely under H0 than under H1. Conversely, a Bayes factor of BF_01_ = 6 suggests that the data are six times more likely under *H*0 than under H1 (for interpretation guidelines on specific Bayes factor values, see Wagenmakers et al., 2011).

In the sequential Bayes factors procedure, data are monitored continuously as they are collected. After testing an initial set of participants, their data are analyzed, and a Bayes factor is calculated. Data collection is finalized if the Bayes factor reaches a pre-defined threshold favoring either H0 or H1, and the corresponding hypothesis is accepted. If neither threshold is reached, the sample size is incrementally increased until it is. A maximum sample size, or stopping rule, is also established to prevent the procedure from requiring an excessive sample size – due to the Bayes Factor fluctuating between H0 and H1 thresholds. In our case, we opted for 200 as a stopping rule (further details are outlined below).

Given the results of Experiment 1, two possible outcomes are conceivable: One possible outcome would posit a parallel influence of the group factor on both happy and fearful expressions. Specifically, the priming scores of happy and fearful ingroup faces would be greater than those of happy and fearful outgroup faces, respectively. This is the result that we have found for RTs in Experiment 1. This outcome means that emotion and group are independently processed or – in other words: A general prejudice effect can be assessed with the procedure but SMA does not apply to the evaluative priming paradigm, even not in its bona fide version. If this scenario comes true, we would finally find substantial Bayes factors in favor of a group effect on priming scores for happy as well as fearful faces.

The second possible outcome is an influence of the group factor on happy expressions but no or a reversed influence on fearful expressions. In this case, again happy ingroup faces would result in higher priming scores than happy outgroup faces, whereas fearful ingroup faces would yield comparable or lower priming scores than fearful outgroup faces. This outcome would support the SMA hypothesis. This is what we found for errors and IES in Experiment 1. If this scenario comes true, we would finally find a substantial Bayes factor in favor of a group effect on the priming scores for happy faces but a substantial Bayes factor in favor of the null hypothesis that there is no larger priming score for fearful ingroup faces compared to fearful outgroup faces.

Thus, our sequential recruitment strategy was to continuously monitor the Bayes factors for the group effect on priming scores for both happy and fearful faces and to stop recruitment when both BFs – that is, the one related to the group effect for happy faces and the one related to the group effect for fearful faces – either exceed a criterion value in favor of the H1 that there is a group effect or exceed a criterion value in favor of the H0 that there is no group effect (in the sense of priming score ingroup > priming score outgroup).

Following the recommendations of Schönbrodt et al. (2017), we pre-specified the following parameters: the H1 boundary was set at BF_+0_ = 6 and the H0 boundary at BF_0+_ = 6 (which corresponds to BF_+0_ = 1/6).^7^ For the JZS H1 effect size prior, we used a scale parameter of *r* = 1 (see also Schönbrodt et al.). Both RT and IES were used as dependent variables, and reaching the boundaries with either measure was sufficient to stop data collection.

The initial sample size was set at *n* = 40 participants. Sample size increment units were set to 10 participants and the stopping rule was set at a maximum of 200 participants. The experiment has been pre-registered at https://aspredicted.org/JX9_4PB and the data can be accessed at https://osf.io/4fmev/.

### Method

#### Participants

The Bayes factor criterion (see above) was reached with an effective sample of 122 (49 females, 70 males, three undisclosed genders; *Mdn*_age_ = 27 years, range: 19–35). One hundred thirty participants were recruited through Prolific Academic (www.prolific.co). Following pre-registered outlier criteria, data were excluded for participants who took more than 90 min to complete the experiment (*n* = 3), or who reported a non-German mother tongue and/or migrant background (*n* = 2). Additionally, *n* = 3 participants were excluded from analyses because they were inadvertently allowed to participate in Experiment 2 despite having already participated in Experiment 1.

#### Design, Materials, and Procedure

Design, Materials, and Procedure were identical to Experiment 1.

### Results

For the priming task, pre-registered sequential testing results are first reported, separately for RT and IES. Additionally, for the sake of convenience and to maintain consistency with Experiment 1, conventional analyses are reported subsequently.

#### ICD Task

In the ICD task, participants demonstrated an average accuracy of 91.98%. Thus, participants paid attention to prime faces during the task.

#### Priming Task

Trials with incorrect or missing responses (4.23% of all trials) of the priming task were excluded from the analysis. Additionally, trials with RTs below 150 ms or more than one-and-a-half interquartile ranges above the third quantile within the individual distribution (5.42% of the remaining trials; Tukey, 1977) were also excluded. Table 2 shows RTs, error rates, and IES; Figure 1B shows the mean priming differences.

**Table 2:** Mean response times (in ms), error rates (in %), and inverse efficiency scores as a function of Emotion, Group, and Target Valence of Experiment 2

Prime		Target	
Emotion	Ethnicity	Negative	Positive
Response times			
Happiness	Ingroup	756 (10)	743 (11)
	Outgroup	749 (10)	750 (10)
Fearful	Ingroup	750 (10)	753 (11)
	Outgroup	745 (9)	763 (11)
Error rates			
Happiness	Ingroup	5.08 (0.52)	3.16 (0.35)
	Outgroup	4.73 (0.42)	3.67 (0.35)
Fearful	Ingroup	4.65 (0.39)	4.41 (0.39)
	Outgroup	3.77 (0.37)	4.41 (0.39)
Inverse efficiency scores (IES)			
Happiness	Ingroup	799 (11)	769 (12)
	Outgroup	789 (11)	780 (11)
Fearful	Ingroup	788 (11)	791 (13)
	Outgroup	776 (11)	799 (11)

##### Sequential Testing (Pre-Registered Analysis)

**Figure 2:**
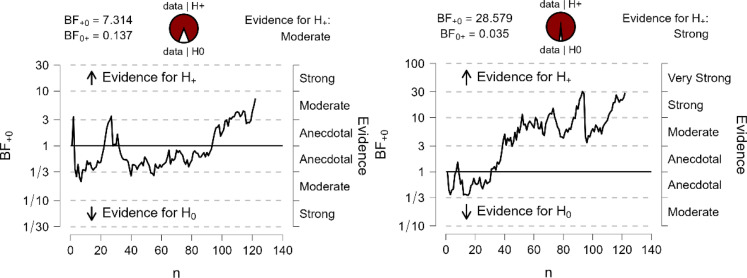
The evolution of the Bayes factors for the group effect with RTs as the dependent variable, with the graph for happy expressions on the left and the graph for fearful expressions on the right, illustrating the trajectory of the Bayes factor (BF_+0_) as participants are incrementally collected. *Note*. The *x*-axis represents the cumulative number of participants, while the *y*-axis shows the corresponding Bayes factor values.

The effective sample revealed evidence favoring one of our pre-defined outcomes using directed Bayesian *t*-tests. For RT, happy ingroup primes (*M* = 13 ms, *SD* = 58) yielded higher priming scores than happy outgroup primes (*M* = −1, *SD* = 54; BF_+0_ = 7.31; *d*_*Z*_ = 0.26). Similarly, fearful ingroup primes (*M* = −3 ms, *SD* = 50) produced higher priming scores than fearful outgroup primes (*M* = −17, *SD* = 48; BF_+0_ = 28.58; *d*_*Z*_ = 0.30). The Bayes factor provided ‘substantial’ and ‘strong’ evidence (see Wagenmakers et al., 2011) for the *H*1 for happy and fearful expressions, respectively (see Figure 2). Given that both Bayes factors exceeded our criterion value of 6, we decided to terminate data collection at this point. Both happy and fearful expressions of ingroup primes yielded higher priming scores than happy and fearful outgroup primes.

For the sake of transparency, priming scores based on IES were consistent with the RT results. Happy ingroup primes (*M* = 30 ms, *SD* = 93) again yielded higher priming scores than happy outgroup primes (*M* = 9 ms, *SD* = 74; BF_+0_ = 5.21; *d*_*Z*_ = 0.25), and fearful ingroup primes (*M* = −3 ms, *SD* = 80) produced higher priming scores than fearful outgroup primes (*M* = −23 ms, *SD* = 75; BF_+0_ = 12.31; *d*_*Z*_ = 0.30).

##### Conventional Statistics (Not Preregistered)

Conventional 2 (prime emotion: happy, fear) × 2 (prime group: ingroup, outgroup) repeated measures ANOVA with RT, error rates, and IES priming scores as the dependent variable were conducted separately.

##### Reaction Times

The analysis revealed significant main effects for both emotion, *F*(1,121) = 28.26, *p* < .001, η_*p*_^2^ = 0.189, *d*_*Z*_ = .48, and group features *F*(1,121) = 18.69, *p* < .001, η_*p*_^2^ = 0.134, *d*_*Z*_ = 0.39. The priming score for happy faces was larger than the priming score for fearful faces. Similarly, ingroup primes produced higher priming scores than outgroup primes. However, no significant interaction was observed, *F*(1,121) = 0.01, *p* = .941, η_*p*_^2^ < 0.001, *d*_*Z*_ = 0.01.

##### Error Rates

A significant main effect of emotion was observed, *F*(1,121) = 14.02, *p* < .001, η_*p*_^2^ = 0.104, *d*_*Z*_ = 0.34, alongside a significant effect for group, *F*(1,121) = 3.54, *p* = .062, η_*p*_^2^ = 0.028, *d*_*Z*_ = 0.17. The priming score for happy faces was larger than the priming score for fearful faces. Additionally, ingroup primes yielded a higher priming score than outgroup primes. However, no evidence of an interaction was found, *F*(1,121) = 0.001, *p* = .978, η_*p*_^2^ < 0.001, *d*_*Z*_ = 0.01.

##### Inverse Efficiency Scores

The result demonstrated a significant main effect of emotion, *F*(1,121) = 33.68, *p* < .001, η_*p*_^2^ = 0.218, *d*_*Z*_ = 0.53, as well as a significant main effect of group, *F*(1,121) = 15.27, *p* < .001, η_*p*_^2^ = 0.112, *d*_*Z*_ = 0.35. The priming score for happy faces was larger than the priming score for fearful faces. Furthermore, ingroup primes resulted in a higher priming score than outgroup primes. Yet again, no evidence of interaction was observed, *F*(1,121) = 0.01, *p* = .923, η_*p*_^2^ < 0.001, *d*_*Z*_ = 0.01.

### Discussion

Experiment 2 aimed to find conclusive evidence for independent or interactive processing of emotion and ethnicity features in the bona fide version of the EPT. The results are clear: the prediction of a main effect of ethnicity was supported, indicating that ingroup primes elicited higher priming scores for both happy and fearful expressions compared to outgroup primes. There was no support for the SMA. We will discuss the implications of these results.

## General Discussion

The present study builds on the puzzling findings of previous EPTs, which reported both independent (Craig et al., 2014; Paulus & Wentura, 2018) and interactive (Weisbuch & Ambady, 2008) effects of emotion and group features. Studies showing independent influences found that emotional expression had a strong impact, while the group factor had a weaker or negligible effect unless it was made task-relevant. Specifically, when the salience of the group factor was increased through tasks that explicitly mentioned group membership (e.g., by classifying group membership of primes in a pre-task or reporting group membership after responding to targets in the EPT), this led to an independent group main effect (e.g., Craig et al., 2014; Paulus & Wentura, 2018). However, in their initial study, Weisbuch and Ambady (2008) reported interactive priming effects, which – so far – have been conceptually replicated using other paradigms (i.e., approach/avoidance, Paulus & Wentura, 2014; the extrinsic affective Simon task, Gurbuz et al., 2023). This mixed evidence raised the question of whether interactive effects can emerge in an EPT when specific processing conditions are established. To investigate this, we conducted two experiments where we increased the salience of the group factor through a secondary task (i.e., ICD, see “Procedure” of Experiment 1) without explicitly mentioning group membership. Experiment 1 revealed independent influences of emotion and group features: Happy and ingroup primes resulted in higher RT priming scores than fearful and outgroup primes, respectively. Priming scores for error rates and inverse efficiency scores, however, yielded some hint of an interactive pattern: Whereas happy ingroup primes again yielded higher priming scores than outgroup happy primes, no difference was observed between the priming effects of fearful ingroup and outgroup primes.

To finally clarify the issue, we conducted Experiment 2, which showed a clear-cut result demonstrating that emotion and group features in the EPT tend to yield independent effects. No hint of an Emotion **×** Group Interaction was found in Experiment 2.

Nevertheless, it should be noted that the introduction of the ICD task into the evaluative priming task yielded a replicable prejudice-related effect: Outgroup primes (relatively) tended to trigger negative responses whereas ingroup primes (relatively) tended to trigger positive responses. Note that the ICD task only required participants to attend to the prime stimuli (to determine if the probe faces were the same identity as the primes); group membership, however, was never explicitly mentioned. Of course, since we did not conduct an experiment including a factor ICD present versus ICD absent, we cannot be sure that the introduction of the ICD really increases the processing of the group feature. However, given finally Experiment 2 we can be rather sure that it does not increase the integrated processing of the two features (i.e., emotion and group).

Post hoc, one might even argue that the ICD might have prevented the integrated processing of emotion and group since the emotional expressions – i.e., happiness or fear – always changed into neutral from prime to probe faces. Thus, strategically it would have been beneficial for ICD performance to discount the emotional expression of the prime.^8^

The puzzle of the EPT remains: Why does the EPT show (predominantly) independent processing of the two features, whereas other paradigms assessing involuntary evaluations show interactive processing (e.g., Gurbuz et al., 2023; Paulus & Wentura, 2014)? And why does the EPT sporadically (Weisbuch & Ambady, 2008, Paulus & Wentura, 2018, Exp. 2, the present Experiment 1) also show interactive effects?

We think that by adopting a certain perspective on the paradigm, we can somehow reduce the “mystery” of the problem. We propose that different prime-related processes occur with different probabilities across trials. Because of the brief exposure and the task-irrelevance of the stimulus, it is generally plausible – even for the standard version of the EPT – to assume that the valence of the prime is processed only in a subset of trials, that is, only with a certain probability. If a prime is characterized by two features, we can plausibly assume that on any given trial, either none of the features, only feature A (e.g., emotion), only feature B (e.g., ethnicity), or both features are processed. Of course, priming effects depend on which feature(s) are currently being processed. In the given context, one might reconstruct from the results that the baseline probability of (sole) emotion processing is rather high, which produces an emotion priming effect. (Sole) ethnicity feature processing seems less likely but still leads to an overall prejudice priming effect (Paulus & Wentura, 2018; Craig et al., 2014, with a race-focused task; the present experiments). Only when both features are processed in a given trial is there a chance of integration, possibly leading to SMA-like moderations.

This reconstruction in terms of different base probabilities of specific processes might remind readers of similar argumentations in memory research (e.g., Buchner et al., 2009), leading to the application of multinomial processing tree models (MPT; e.g., Hu & Batchelder, 1994). In the online supplement to this article (see https://osf.io/4fmev/), we suggest and test an MPT model that provides further support for these assumptions by estimating parameters for emotion processing, ethnicity processing, and the processing of the compound of emotion and ethnicity. This reconstruction has the potential to shed some light on the EPT puzzle. In this regard, Experiments 1 and 3 of Paulus and Wentura (2018), the experiments of Craig et al. (2014), and our Experiment 2 are dominated by large probabilities of (only) emotion processing and low to moderate processing of (only) ethnicity, but a negligible probability of integrated processing. Post hoc, one could argue that in Paulus and Wentura's Experiment 2 and as well as in our Experiment 1, the probability of integrated processing was large enough to lead to an overall SMA-like interaction pattern. Of course, we acknowledge that have yet to determine how to reliably increase the probability of integrated processing. It appears that interactive processing requires additional conditions, and the introduction of the identity change task was not the right candidate. Despite these challenges, the present study adds new insights into the puzzling effects observed in the EPT. We encourage researchers to follow up on this route, as it means that prejudice-related processing can be malleable, though identifying the most effective strategies remains a challenge.

## Footnotes

^1^
A priming effect (i.e., faster responses on congruent trials compared to incongruent trials) emerged when happy ingroup and fearful outgroup faces were perceived as congruent to positive targets (and as incongruent to negative targets), whereas happy outgroup and fearful ingroup faces were perceived as congruent to negative targets (and incongruent to positive targets). Thus, strictly speaking, statements such as “*x* facilitated reactions to positive targets” and “*y* facilitated reactions to negative targets” cannot be independently tested (see, e.g., Wentura & Degner, 2010). Nevertheless, we have chosen to retain this wording because it is more reader-friendly; the word “relatively” is intended to recall the limitation explained in this footnote.

^2^
Negative: gierig (greedy), grausam (cruel), boshaft (malicious), gemein (mean), geizig (stingy), aggressiv (aggressive), kriminell (criminal), autoritär (authoritarian), brutal (brutal), treulos (disloyal); positive: human (humane), ehrlich (honest), gütig (kind), gerecht (just), gedulding (patient), sanft (gentle), humorvoll (humorous), tolerant (tolerant), friedlich (peaceful), aufrichtig (sincere).

^3^
Two participants performed around 50% accuracy (i.e., chance level). However, excluding their data did not make any difference to the essential results.

^4^
This interaction can be regarded as one-tailed significant when considering our prediction regarding the interaction (i.e., happiness is expected to yield higher priming scores for the ingroup compared to the outgroup, while fear is anticipated to result in higher priming scores for the outgroup compared to the ingroup). It is important to note that *F*-tests with 1 degree of freedom in the numerator are equivalent to *t*-tests, allowing for the use of one-tailed significance testing (see Maxwell & Delaney, 2004, p. 164).

^5^
This statement holds for RT priming scores as well, *t*(87) = 1.77, *p* = .080, *d*_*Z*_ = .19. (To compare, for happy faces: *t*(87) = 2.95, *p* < .004, *d*_*Z*_ = .31.).

^6^
To compare to the present Experiment 1, a reanalysis shows that the priming score for ingroup happiness significantly exceeded that for outgroup happiness, *t*(82) = 4.10, *p* < .001, d*Z* = .45, whereas ingroup and outgroup fear were comparable in terms of mean priming scores, *t*(82) = 1.60, *p* = .113, d*Z* = .18.

^7^
The notation BF_+0_ (instead of BF_10_) indicates a Bayes factor referring to a directed *H*1 hypothesis (i.e., here: ingroup faces would result in higher priming scores than outgroup faces). Accordingly, BF_0+_ indicates a Bayes factor referring to a directed *H*0 hypothesis (i.e., here: ingroup faces would *not* result in higher priming scores than outgroup faces); thus, not only a null result but also a reversed effect (i.e., here: ingroup faces would result in a lower priming score than outgroup faces) would lead to a high BF_0+_.

^8^
We thank an anonymous reviewer for pointing out this problem.

## Notes

**Publication Ethics:** Our studies did receive approval from the research ethics committee of the faculty of human and business sciences of Saarland University, Germany.

**Authorship:** Emre Gurbuz, conceptualization, data curation, formal analysis, investigation, methodology, project administration, resources, software, supervision, validation, visualization, writing – original draft. Michaela Rohr, methodology, writing – review & editing. Dirk Wentura, conceptualization, formal analysis, funding acquisition, methodology, project administration, supervision, writing – review & editing. All authors approved the final version of the article.

**Open Science:** Open Data, Open Materials: The data needed to reproduce all of the reported results, as well as materials used to conduct the study are available at https://osf.io/4fmev/ (Gurbuz et al., 2024).

Pre-registration: Experiment 1 has been pre-registered at https://aspredicted.org/56R_NYH.
